# *Complexity Variability* Assessment of Nonlinear Time-Varying Cardiovascular Control

**DOI:** 10.1038/srep42779

**Published:** 2017-02-20

**Authors:** Gaetano Valenza, Luca Citi, Ronald G. Garcia, Jessica Noggle Taylor, Nicola Toschi, Riccardo Barbieri

**Affiliations:** 1 Massachusetts General Hospital/Harvard Medical School, Boston, MA, USA; 2 Department of Information Engineering and Bioengineering and Robotics Research Centre “E. Piaggio”, School of Engineering, University of Pisa, Italy; 3 School of Computer Science and Electronic Engineering, University of Essex, Colchester, UK; 4 Masira Research Institute, School of Medicine, Universidad de Santander, Bucaramanga, Colombia; 5 Nell Hodgson Woodruff School of Nursing, Emory University, Atlanta, GA, USA; 6 University of Rome “Tor Vergata”, Rome, Italy; 7 Politecnico di Milano, Milan, Italy

## Abstract

The application of complex systems theory to physiology and medicine has provided meaningful information about the nonlinear aspects underlying the dynamics of a wide range of biological processes and their disease-related aberrations. However, no studies have investigated whether meaningful information can be extracted by quantifying second-order moments of time-varying cardiovascular complexity. To this extent, we introduce a novel mathematical framework termed *complexity variability*, in which the variance of instantaneous Lyapunov spectra estimated over time serves as a reference quantifier. We apply the proposed methodology to four exemplary studies involving disorders which stem from cardiology, neurology and psychiatry: Congestive Heart Failure (CHF), Major Depression Disorder (MDD), Parkinson’s Disease (PD), and Post-Traumatic Stress Disorder (PTSD) patients with insomnia under a yoga training regime. We show that complexity assessments derived from simple time-averaging are not able to discern pathology-related changes in autonomic control, and we demonstrate that between-group differences in measures of complexity variability are consistent across pathologies. Pathological states such as CHF, MDD, and PD are associated with an increased *complexity variability* when compared to healthy controls, whereas wellbeing derived from yoga in PTSD is associated with lower time-variance of complexity.

Physiological dynamics associated with oscillatory systems (such as the cardiovascular system) are commonly characterized through mathematical approaches in both the time and frequency domains. Most of these approaches assume intrinsic linearity and time-invariant properties. The inherent postulate is that the magnitude of physiological responses is proportional to the strength/amplitude of the input stimuli. Given the widespread accessibility of electrocardiographic (ECG) as well as pulseoximeter measurements, the analysis of Heart Rate Variability (HRV) has become a paradigmatic example of physiological time series analysis performed through linear techniques. HRV analysis is commonly based on indices such as mean heart rate, standard deviation, and low-frequency (LF) and high-frequency (HF) spectral powers derived from the RR interval series^1^. However, the cardiovascular system is constantly involved in a dynamical, mutual interplay with numerous other physiological subsystems (e.g., endocrine, neural, and respiratory), as well as in multiple self-regulating, adaptive biochemical processes^2^,^3^,^4^. In this context, it is well known that the effects of combined sympathetic and vagal stimulation on heart rate are not simply additive, as tonic sympathetic stimulation sensitizes the heart rate to vagal stimulation^4^. This is because sympathetic stimulation inhibits acetylcholine release by acting on adrenergic receptors on the vagal terminals, cytosolic adenosine 3,5-cyclic monophosphate mediates postjunctional interactions between the sympathetic and vagal systems, and acetylcholine released by vagal stimulation inhibits norepinephrine release by acting on muscarinic receptors on sympathetic nerve terminals. In addition, neuropeptide Y released from sympathetic nerve terminals also interacts with ACh acetylcholine, and the release of neuropeptide Y is prevented by simultaneous vagal stimulation^4^. As a results, cardiovascular dynamics exhibits an inherently complex structure characterized by non-stationary, intermittent, scale-invariant and nonlinear behaviors^1^,^5^.

In light of the above, methodological approaches derived from the theory of complex dynamical systems may provide access to a more complete description of the mechanisms underlying biological regulation of cardiac activity. Widely employed methods for characterizing heartbeat complexity include detrended fluctuation analysis and wavelet analysis (which quantify scaling properties, correlations, and fractal measures of variability), Lyapunov exponents as well as various measures of entropy such as sample entropy and its multiscale version (which quantify the degree of instability and predictability of the time series under investigation)^6^,^7^,^8^,^9^. The use of these methods has allowed improved characterization of abnormal cardiac rhythms^1^,^10^,^11^ and has aided in predicting the risk of acute adverse events such as sudden cardiac and sudden infant death (see refs 1,9, 10, 11, 12, 13, 14, 15, 16).

## Current Limitations in Complexity Assessment

Despite the considerable achievements obtained by the measures and approaches outlined above, the application of these analysis strategies to physiological systems has resulted in several discrepancies in the literature. For example, changes in cardiovascular complexity have been observed to accompany aging^17^, whereas other findings suggest that fractal linear and nonlinear characteristics of cardiovascular dynamics do not change with age^18^. Similar controversies have been reported in the field of sleep analysis^19^. In this paper, we posit that these discrepancies may partly be due to several methodological and applicative issues inherent to these methods, which have not yet been satisfactorily addressed.

First, the intrinsically discrete nature of heartbeats, which are unevenly spaced in time, often leads to the use of interpolation procedures, which are likely to introduce bias in the estimation of nonlinear/complexity measures. Second, traditional complexity estimation approaches/algorithms provide a single value (or set of values) within a predetermined time window and hence can only represent average measures of the physiological system dynamics observed in the entire time window. However, it is well known that physiological dynamics commonly undergo rapid, transient changes in time which can also occur in a number of psycho-physiological states and pathologic conditions^20^,^21^,^22^,^23^. In the face of non-stationary behavior, collapsing across time into a single, more or less representative value may not allow to capture the subtleties of complex behavior within any particular analysis window. Moreover, even in case of windowing strategies that would allow for the computation of more than one reference point estimation, this may not be sufficient to properly catch the time-varying dynamics of the computed measure. Third, most of the nonlinearity and complexity measures employed to-date have been proven to be sensitive to the presence of uncorrelated (e.g. white) or correlated (e.g. 1/*f*) noise. As stochasticity plays a crucial role in physiological dynamics^6^,^10^, this sensitivity may lead to an overestimation of complexity which may become more evident in the presence of specific pathologies, such as certain cardiac arrhythmias including atrial fibrillation^24^. While when compared to healthy systems, these pathological situations appeared to be associated with the emergence of a more regular cardiovascular behavior(visible as a reduction in entropy)^11^, it was shown that the observed changes were due to modifications in the statistical properties of underlying physiological noise^24^.

## A New Time-varying Model for Complexity Assessment

To overcome these limitations, we recently introduced novel time-varying complexity measures that can be applied to stochastic discrete series such as the ones related to heartbeat dynamics^7^,^8^. These novel measures are fully embedded in the probabilistic framework of the inhomogeneous point-process theory and are obtained by modeling the cardiovascular system through both deterministic and random terms. In turn, this caters for the simultaneous presence of both chaotic and stochastic behaviours. This idea has been successful applied in other studies^6^,^25^, and is in agreement with current views on the genesis and physiology of healthy heartbeat dynamics, which can be thought of as the output of a nonlinear deterministic system (the pacemaker cells of the sinus node) forced by a high-dimensional input (neural activity of fibers innervating the sinus node itself). The originality of the new definitions lies in the explicit mathematical formulations of the time-varying phase-space vectors, as well as in the definition of their distance^7^,^8^.The main advantages of these techniques are that the resulting, instantaneous estimates of complexity are free from bias due to either interpolation techniques or variability in statistical properties of noise.

## Complexity Variability

Once instantaneous complexity series are available, basic time-domain features can be used to summarize cardiovascular complex dynamics. In particular, measures of central tendency, e.g., the median value, and variability, e.g., the median absolute deviation can be calculated.

The former (central tendency) can be considered equivalent to standard complexity estimates which collapse data across time by design. The latter (median absolute deviation) represents an innovation in the field of complexity analysis^7^,^8^, by defining a measure of *complexity variability*.

In this context, when adopting instantaneous Lyapunov exponents as a complexity measure^7^, we recently observed a notable (albeit preliminary) discriminant power associated with its variability. In a recent study on patients with congestive heart failure (CHF) and healthy subjects^24^ we found that neither standard sample (SampEn) and approximate (ApEn) entropy measures^1^,^26^ nor the median (over a given time-window) of the instantaneous dominant Lyapunov exponent are able to discriminate between the two populations. In contrast, heartbeat dynamics associated with CHF showed a significantly increased complexity variability when compared to healthy controls, hence providing a novel measure which could potentially aid in early discrimination and/or stratification of this kinds of patients^27^,^28^. Of note, these findings are in accordance with current literature indicating an effect of cardiovascular disorders on complexity and variability of biological processes^29^.

## Novel Definitions and Applications of Complexity Variability

In this study, we hypothesize that the discriminative potential of complexity variability measures can serve as a potential biomarker able to discriminate subtle changes which are not evident in other complexity measures. To this end, in this study we aimed to broaden the spectrum of pathologies under study to patients suffering from neurological and mental disorders such as major depression disorder (MDD), Post Traumatic Stress Disorders (PTSD), and Parkinson’s Disorders (PD). In the rest of this paper, prior art concerning cardiovascular assessment of these pathologies is reported. Then, the basic mathematical formulation of inhomogeneous point-process models of heartbeat dynamics, as well as of instantaneous Lyapunov estimates are reported followed by experimental results, discussion and conclusion.

## Heartbeat Dynamics in Cardiovascular, Mental and Neurological Disorders

### HRV Assessment in Congestive Heart Failure

Congestive Heart failure (CHF) is a major public health problem, with a prevalence of more than 5.8 million in the United States and more than 23 million worldwide^30^. HRV analysis has been previously used to discern healthy subjects from patients suffering from congestive heart failure (CHF)^31^,^32^,^33^,^34^,^35^,^36^,^37^. It has been accepted that linear features of heartbeat dynamics (based on spectral analysis) are not sufficient for CHF patient characterization, and need to be complemented by nonlinear features, ranging from Entropy to Non-Gaussian metrics (see refs 7,8,23,31, 32, 33,37,38 and reference therein for reviews). Also, classic approximate and sample entropy, as expressed in their basic form, are not able to discern between heathy subjects and patients with CHF^8^,^24^.

Moreover, cardiovascular dynamics in CHF patients was associated with a loss of multifractality, whose information is encoded in the Fourier phases of HRV series^23^,^32^,^33^,^37^. Furthermore, in CHF patients, departures from Gaussianity have been used to evaluate increased mortality risk^34^,^35^,^38^.

### HRV Assessment in Major Depression

According to epidemiological studies, almost 15% of the population in the United States has suffered from at least one episode of mood alteration^39^, and about 27% (equals 82.7 million; 95% confidence interval: 78.5–87.1) of the adult European population is or has been affected by at least one mental disorder^40^. To date, biological markers, especially those derived from applying advanced signal processing approaches to biological signals, are not commonly incorporated in clinical routine examinations^41^,^42^. Previous studies have focused on depression and sleep^43^,^44^ and circadian heart rate rhythms^45^,^46^ highlighting autonomic changes that may be considered predictors of clinical modifications. In the realm of HRV analysis, a decrement of HF power and an increment of LF/HF ratio was observed in MD patients when compared to controls^47^. However, several studies demonstrated that estimates of linear cardiovascular dynamics, i.e., quantifiers of the power distribution among frequencies only, are unable to adequately discern healthy subjects from MD patients^22^,^43^,^48^,^49^,^50^,^51^,^52^,^53^.

Nonlinear analysis of HRV data, which also quantifies nonlinear interactions among frequencies reflecting underlying ANS dynamics, represents a recent frontier in the assessment of psychiatric disorders. In this context, nonlinear measures have already allowed the discrimination of depressive patients from healthy subjects, consistently showing a significant decrease of complexity in the pathological cohort^22^,^49^,^50^,^51^. These findings support the hypothesis that complexity of physiologic signals could be used as dynamical biomarkers of depression.

### HRV Assessment in Post Traumatic Stress Disorder with Insomnia

Sleep disturbances and insomnia related to post-traumatic stress disorder (PTSD) are a prototypical example of the comorbidity between autonomic dysfunction psychological distress. Among American adults, the estimated lifetime prevalence of PTSD is 6.8%^54^. Sleep disturbances such as insomnia and nightmares have much higher prevalence (up to 60%) in people with PTSD compared to those without PTSD^55^. As separate conditions, both PTSD and insomnia are characterized by chronic hyperarousal of Autonomic Nervous System (ANS) activity^56^, i.e., high sympathetic and hypothalamicpituitaryadrenal activity), and drug-nave subjects with PTSD display decreased cardiac vagal control when compared to subjects without PTSD and matched controls^57^. Clinically, this overlap is reflected in an entire cluster of the DSM-IV-TR diagnostic criteria for PTSD pertaining to hyperarousal^58^. Accordingly, the DSM-IV-TR PTSD hyperarousal cluster includes assessment of insomnia symptoms, and autonomic dysregulation has also been proposed as an important mechanism in the pathogenesis of insomnia^59^. While we are not aware of literature on ANS function in PTSD-related insomnia, one study suggested the existence of a relationship between sleep disturbances and baroreceptor sensitivity in women with PTSD^60^.

Among the approaches thought to aid in stress reduction and the prevention of mental disorders, yoga has been seen to be an effective strategy^61^. Mixed evidence suggests that yoga influences HRV dynamics in people without PTSD, including advanced yoga practitioners as well as adults exposed to acute trauma and chronic stress^62^,^63^,^64^. While some studies have shown that yoga reduces psychological symptoms in PTSD, no studies have directly investigated HRV dynamics in PTSD patients (with or without insomnia) who practice yoga^65^,^66^. Because insomnia and PTSD involve ANS dysregulation, and because yoga may balance ANS function, HRV analysis has the potential to serve as a biomarker to assess the therapeutic effect of yoga in reducing hyperarousal in PTSD.

### HRV Assessment in Parkinson’s Disorders

Parkinson’s disease (PD) is the second most common neurodegenerative disorder after Alzheimer’s disease, and is classically associated with motor symptoms including tremor, balance problems, limb rigidity, bradykinesia and gait abnormalities^67^. The causes and aetiology of this disease are still largely unknown. Symptoms of ANS failure are known to be part of the disease^68^. They include cardiovascular, sexual, bladder, gastrointestinal, and sudo-motor abnormalities^69^, and previous studies reported a variable prevalence of cardiovascular autonomic dysfunction between 23% and 80%^70^,^71^.

HRV measures have been employed to non-invasively explore ANS alterations in PD by evaluating the modulatory effects of ANS dynamics on sinus node activity^72^. In one of these studies, all HRV spectral components (calculated from studying 24 h outpatient ECG recordings) were found to be significantly lower in the PD patients when compared to control subjects^73^. In another study on 10 minutes of data recorded at rest, HRV-HF power was significantly lower in untreated patients with PD with respect to healthy controls, whereas nonlinear HRV analysis based on entropy and geometrical measures was not able to distinguish between patients and controls^74^. However, PD patients displayed an increase in complexity of systolic arterial pressure series when compared to controls^75^. Taken together, these findings point towards a possible role of HRV analysis characterizing subtle autonomic alterations which accompany major motor symptoms in PD.

## Experimental Setup and Results

In order to validate the *complexity variability* framework, in this paper we pooled four experimental datasets involving cardiovascular, neurological, and mental disorders such as Congestive Heart Failure (CHF), Major Depression Disorder (MDD), Parkinsonos Disease (PD), and Post-Traumatic Stress Disorder (PTSD) with insomnia. Within the CHF, MDD, and PD datasets the patient population was compared with age- and gender-matched healthy controls. In the PTSD dataset, we performed paired comparison of data gathered before and after all patients underwent yoga practice training. Details on each experimental setup follow below.

All features were instantaneously calculated with a *δ* = 5 ms temporal resolution from each recording of each subject. KS and autocorrelation plots were visually inspected to check that all points of the plot were within the 95% of the confidence interval, hence guaranteeing the independence of the model-transformed intervals^76^. NARL model order selection was performed by choosing orders that minimize KS distances (the smaller the KS distance, the better the model fit). Once the order p,q is determined, the initial NARL coefficients are estimated by the method of least squares^76^. Accordingly, our analysis indicated *p* = 3~5 and *q* = 1~3 with *α* = 0.2 as optimal choice.

### Complex Dynamics in Congestive Heart Failure patients

This dataset was selected from data gathered from CHF patients and reference healthy subjects on a public source: Physionet (http://www.physionet.org/)^77^. All participants received information about the study procedures and gave written informed consent approved by the local Institutional Review Board. The experimental protocol was approved by the Hospitals’ Human Subjects Committees. Data were acquired in accordance with the approved guidelines^78^. RR time series were recorded from 14 CHF patients (from BIDMC-CHF Database) as well as 16 healthy subjects (from MIT-BIH Normal Sinus Rhythm Database). Each RR time series, extracted from the 20 h recording at the same day cycle, was artifact-free (upon visual inspection and artifact rejection based on the point-process model^79^) and lasted about 50 min. These recordings have been employed in multiple landmark studies of complex heartbeat interval dynamics^7^,^8^,^12^,^15^,^27^,^80^.

#### Results

**Figure 1:**
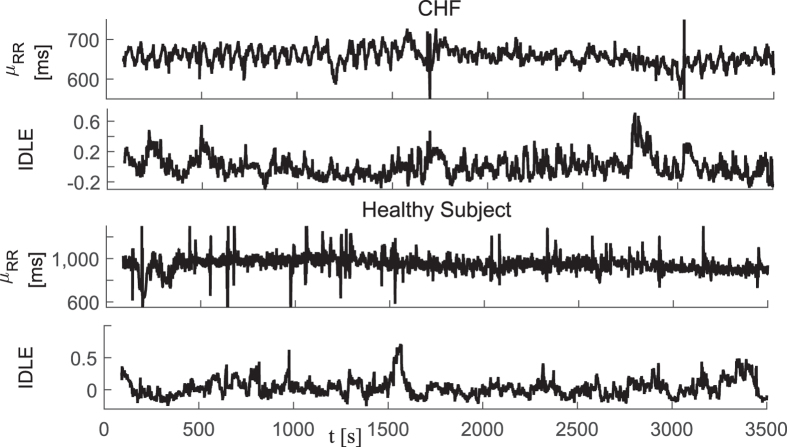
Instantaneous heartbeat statistics computed using a NARL model from a representative CHF patient (top panels) and healthy subject (bottom panels). Estimated *μ*_*RR*_(*t*) and IDLE series are reported.

In this dataset, we tested the ability of instantaneous linear and complex nonlinear estimates of heartbeat dynamics to discriminate healthy subjects from CHF patients. Exemplary instantaneous tracking of complex heartbeat dynamics, along with the first-order moment, are shown in Fig. 1. Group statistics are reported in Table 1. The difference was expressed in terms of p-values calculated through a non-parametric Mann-Whitney test under the null hypothesis that the medians of the two sample groups were equal.

On average, CHF patients show significantly lower *μ*_RR_, *σ*_RR_, as well as lower LF and HF power. The median IDLE (*IDLE*) was not significantly different between the two groups. Conversely, the complexity variability measure, *CV*_*IDLE*_, showed significant statistical difference (*p* < 0.05). It is worth noting that we detected an increase in *CV*_*IDLE*_ in the pathological state (as compared to controls), contrarily to classical nonlinear-based assessments^7^,^8^,^23^,^31^,^32^,^33^,^38^, in which pathology is consistently associated with a decrease of complexity. Our novel quantifier therefore provides an additional dimension associated with incremental knowledge about changes in cardiovascular complexity in CHF^7^,^8^,^23^,^31^,^32^,^33^,^37^,^38^.

### Complex Dynamics in Major Depression Disorder Patients

48 outpatients (age: 22.6 ± 4.7 years) with Major Depression Disorder (MDD) were recruited through screening in a population of University students by applying the Zung-self-rating depression scale^81^. All patients were experiencing their first MDD episode and had not received psychotherapeutic or pharmacological treatment. A control group consisting of 48 age- and gender-matched healthy subjects was also included (age: 23.5 ± 4.9 years). Sixteen men (33.3%) and 32 women (66.6%) were included in each group. Exclusion criteria for both MDD and healthy subjects were: cardio-, cerebro-, or peripheral vascular diseases, the presence of neoplasm, diabetes mellitus, kidney or liver failure, infectious or systemic inflammatory disease and current neurological illnesses. All participants received information about the study procedures and gave written informed consent approved by the local Institutional Review Board of the Cardiovascular Foundation of Colombia, Bucaramanga, Colombia. The experimental protocol was approved by such ethical committee. Data were acquired in accordance with the approved guidelines. Participants abstained from smoking or consuming beverages containing caffeine, xanthines or alcohol the day before evaluation. Continuous ECG monitoring (lead II) was performed with a Finometer device (Finapress Medical System, The Netherlands) while subjects were asked to rest for 10 minutes in a reclining position. Further details can be found in ref. 51.

#### Results

**Figure 2:**
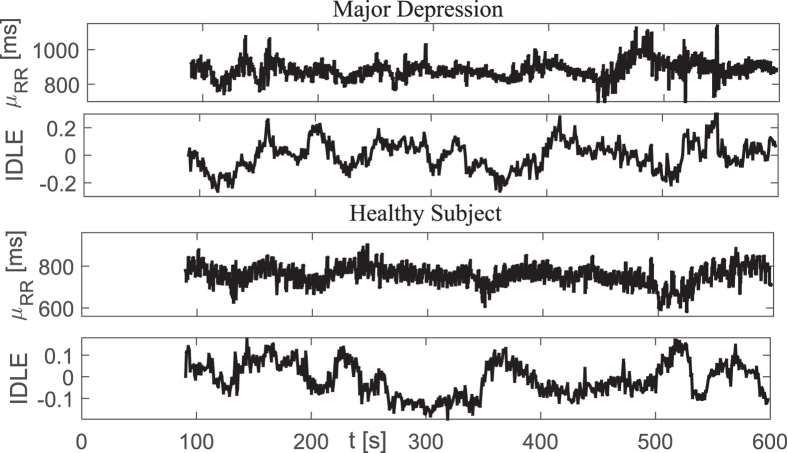
Instantaneous heartbeat statistics computed from a representative MDD patient (top panels) and healthy subject (bottom panels) using a NARL model. Estimated *μ*_*RR*_(*t*) and IDLE series are shown along a 10 minutes of resting state.

Exemplary instantaneous tracking of complex heartbeat dynamics, along with the first-order moment, during 10 minutes of resting state are shown in Fig. 2. Group statistics are reported in Table 2. The difference was expressed in terms of p-values from a non-parametric Mann-Whitney test under the null hypothesis that the medians of the two sample groups are equal.

In summary, in presence of a severe depressive state *CV*_*IDLE*_ provides significant statistical power in discerning MDD from healthy subjects. To our knowledge, this is the first time that a time-varying complexity assessment is proposed in mental disorders. Our results are also in agreement with previous studies demonstrating that estimates of linear cardiovascular dynamics are unable to adequately discern healthy subjects from MD patients^22^,^43^,^48^,^49^,^50^,^51^,^52^,^53^. Also, as in CHF (see above), classical complexity measures have been seen to decrease in MDD when compared to healthy controls^48^,^49^,^53^. In this study, we show that our *CV*_*IDLE*_ measure is significantly increased in MDD as compared to controls, hence providing additional information about complex cardiovascular changes.

### Complex Dynamics in Post-Traumatic Stress Disorder with Insomnia and Yoga Training

The overall objective of this experimental setup was to evaluate the potential of yoga as an adjunctive treatment for insomnia related to PTSD. Nineteen adults (over 18 years-old) were recruited to participate in this study. Insomnia and PTSD unrestricted by trauma history was confirmed in study participants using the Clinician-Administered PTSD Scale and the American Academy of Sleep Medicine’s Research Diagnostic Criteria for insomnia related to a mental health disorder^82^,^83^. All participants received information about the study procedures and gave written informed consent approved by the local Institutional Review Board (Partners Human Research Committee, Brigham and Women’s Hospital, Boston, MA, USA) The experimental protocol was approved by the Institutional Review Board. Data were acquired in accordance with the approved guidelines. Participants were asked to continue any other stable treatments they were on (pharmacological and behavioral) 6 weeks from baseline throughout the duration of the study. Also, all subjects were naive to yoga (<1 hour/week in the past 6 months). The intervention consisted of an 8-week, closed-group yoga program: classes met twice weekly for 90 minutes, with 15-minute personal practice on non-class days guided by DVD or online. This manualized program included ethics, postures, breath regulation, relaxation and basic meditation techniques taught in the Kripalu yoga style. The first 4 weeks focused upon learning all techniques except meditation, while building safety and trust; the second half involved more time in poses and breaths, and introduced meditation. Continuous ECG data was collected at baseline and at end of treatment 5 minutes of resting state with regular breathing. Out of the 19 participants who completed the study (50% attrition), 12 had evaluable baseline and end-treatment ECG data.

#### Results

**Figure 3:**
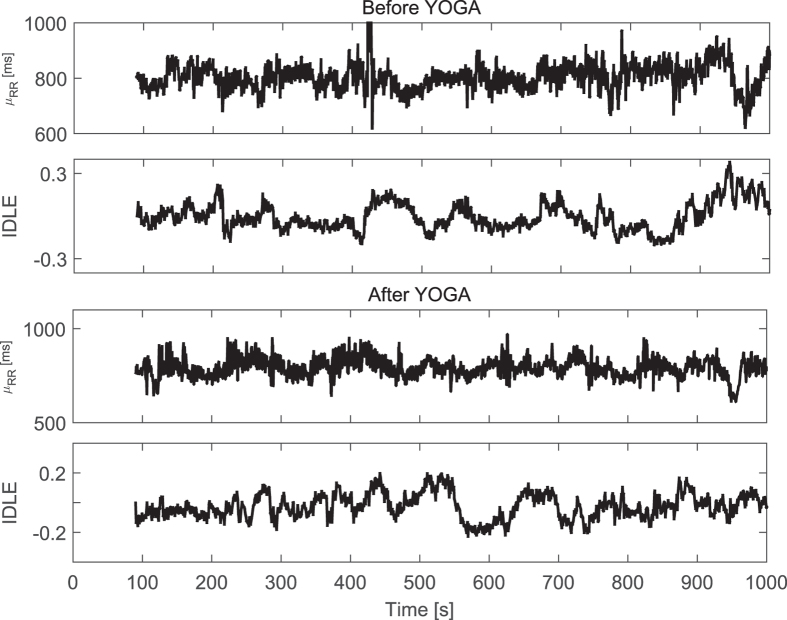
Instantaneous heartbeat statistics computed using a NARL model from a representative PTSD patient before (top panels) and after (bottom panels) performing a yoga training. Estimated *μ*_*RR*_(*t*) and IDLE series are reported.

Instantaneous linear and complex nonlinear estimates of heartbeat dynamics were used to investigate significant changes in ANS activity between before- and after-performing the yoga training. Exemplary instantaneous tracking of the complex heartbeat dynamics, along with the first-order moment are shown in Fig. 3. Paired group statistics are reported in Table 3. The difference was expressed in terms of p-values from a non-parametric Wilcoxon test under the null hypothesis that the medians of the two paired sample groups are equal.

In particular, in PTSD patients who improved their mental well-being by decreasing their psychological distress after yoga training (e.g., PTSD symptoms decreased with *p* < 0.005 from 57.1 ± 2.5 at baseline to 46.8 ± 2.9 based on the PTSD Checklist - Specific for the Diagnostic and Statistical Manual for Mental Health Disorders, 4th edition; from Noggle *et al*., in preparation), our complexity variability measure was significantly lower when comparing data acquired before and after training. To our knowledge, estimates of *CV*_*IDLE*_ are able for the first time to provide an effective quantification of improved mental well-being employing exclusively cardiovascular dynamics.

### Complex Dynamics in Parkinson’s Disease

Cardiovascular signals were recorded from 29 healthy controls (HC, 18 males) and 30 PD patients (23 males). Subjects were placed horizontally in a supine position and remained at rest during the whole recording (10 minutes). During the acquisition, all subjects were instructed not to talk and maintained relaxed spontaneous breathing. All participants gave written informed consent to participate in the study, which was approved by the Versilia Hospital, AUSL 12 Viareggio, Lido di Camaiore (LU), Italy, committee. The experimental protocol was approved by the local ethics committee. Data were acquired in accordance with the approved guidelines. Clinical assessment included history of disease-related symptoms and signs, and full neurological examination. All patients were screened for cardiovascular autonomic dysfunction which was considered as exclusion criterion. All patients had to satisfy the UK Brain Bank criteria for the diagnosis of PD^84^ and were in stage 1, 1.5 2 or 2.5 according to the Hoehn-Yahr (HY) system. As supportive criterion, a 123IFP-CIT SPECT to confirm nigrostriatal degeneration was performed. Severity of parkinsonism was evaluated by the Unified Parkinsonos Disease Rating Scale (UPDRS)^85^ and the HY staging system^86^.

#### Results

**Figure 4:**
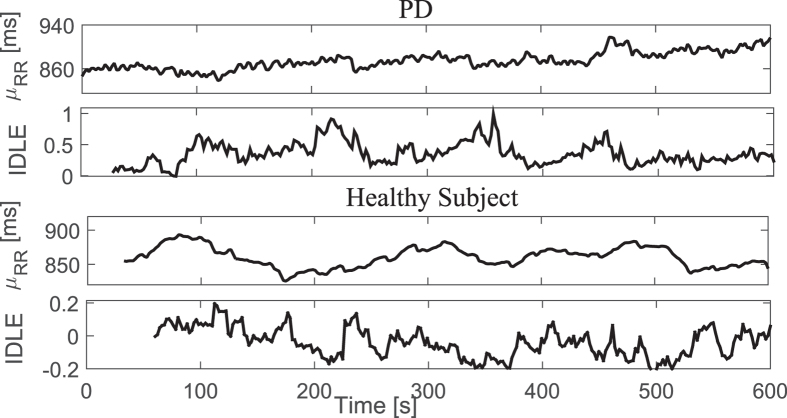
Instantaneous heartbeat statistics computed using a NARL model from a representative PD patient (top panels) and healthy subject (bottom panels) during 10 minutes of resting state. Estimated *μ*_*RR*_(*t*) and IDLE series are reported.

In this case, we tested the ability of instantaneous linear and complex nonlinear estimates of heartbeat dynamics in discriminating healthy subjects from PD patients. Exemplary instantaneous tracking of the complex heartbeat dynamics, along with the first-order moment, during 10 minutes of resting state are shown in Fig. 4. Group statistics are reported in Table 4 (reproduced from^28^ with permission). The difference was expressed in terms of p-values from a non-parametric Mann-Whitney test under the null hypothesis that the medians of the two sample groups are equal.

Importantly, our results suggest that PD states are associated with an increase of complexity variability, possibly pointing toward subtle autonomic changes which may accompany or precede autonomic dysfunctions, and can only be detected using an instantaneous, time resolved approach to quantifying autonomic complexity.

## Discussion and Conclusion

We presented a novel *complexity variability* framework for the assessment of complex physiological dynamics. This work was motivated by the fact that a single complexity estimation, which collapses data across time, may not be sufficient to completely characterize physiological system complexity in the face of non-stationary behavior^20^,^21^,^22^,^23^. Importantly, accounting for temporal dynamics of stochastic events is fundament for the assessment of complex ecosystems, too^87^,^88^.

Our methodological approach is based on the time-varying assessment of Lyapunov exponents (e.g. the IDLE index) within a point-process paradigm which has been explicitly devised for modeling cardiovascular control. We performed instantaneous IDLE estimates in four exemplary datasets, involving data gathered in patients with cardiovascular disease such as CHF, as well as in neurological and psychiatric disorders such as MDD, PTSD with insomnia, and Parkinson’s Disease (PD). The time-varying IDLE information was summarized through measures of central tendency (median value) and variability (median absolute deviation).

In all statistical comparisons of patients vs controls or patients before treatment vs patients after treatment, the central tendency of complexity measures did not show any significant differences. Conversely, our *complexity variability* measures always showed statistically significant differences. Moreover, when compared to other instantaneous heartbeat estimates defined in the time and frequency domains^76^, only heartbeat *complexity variability* measures showed significant differences in the MDD, PTSD and insomnia treatment, and PD datasets.

Of note, in the attempt to provide a plausible comparison set, we were not able to find high time-resolution indices that were going beyond the standard heart rate variability indices (linear and nonlinear). Reasonably, we do not exclude that other indices derived by processing series of heartbeat dynamics might present meaningful characteristics able to effectively discern between, e.g., healthy control subjects and patients with major depression, Parkinson’s disease, congestive heart failure, or post traumatic stress disorder. Indeed, we aimed to study clinically meaningful, statistical properties of complexity variability, i.e., of the variance of a series quantifying the complexity level of a nonlinear system.

While the findings reported in this study will need further characterization before they can be directly translated into clinical practice, the presence of significant differences in complexity variability measures between MDD, PTSD and insomnia treatment, provides an indication that these physiological measurements could potentially aid in the early differential diagnosis between subjects experiencing normal grief or anxiety symptoms and those eventually evolving to clinical depression or PTSD in response to a stressful life event. This is of particular importance for clinical practitioners, given that normal grief, although sharing similar clinical symptoms with depression, neither involves the physiological alterations reported in MDD nor requires pharmacological interventions. Furthermore, consistently detecting altered autonomic features could provide an indication of how the modulation of peripheral physiology by cortical and subcortical pathways becomes disrupted during active MDD and PTSD. The study of complex ANS activity could therefore potentially be explored as an early physiological index for remission or relapse in patients under treatment. Interestingly, in the case of cardiovascular and mental/neurological disorders, complexity variability measures were significantly higher in the pathological groups as compared to controls (healthy subjects). Consistent with these findings, in case of PTSD patients who improved their mental well-being, complexity variability measures were significantly lower when comparing data acquired before and after yoga training. Additionally, we found an increased complexity variability in PD patients when compared to controls. While the etiology of ANS disturbances in PD (also known as dysautonomia) are not yet well-understood, it is known that they reflect neurodegenerative processes which involve additional circuits apart from the nigrostriatal dopaminergic system^89^,^90^, and develop in a manner which is largely independent of the evolution of dopaminergic symptoms. Accordingly, it has been shown that the assessment of cardiovascular autonomic failure can aid in early recognition and treatment of PD^91^,^92^, and previous studies have demonstrated parkinsonisms such as Multiple System Atrophy and Progressive supranuclear palsy display different patterns of disease-related alterations with respect to PD^93^,^94^. A better understanding, early recognition and treatment of ANS failure in PD may therefore aid in the differential diagnosis of Parkinsonisms as well as have an impact on patient management quality and therefore quality of life. In this context, currently the evaluation of ANS dysfunctions relies on combersome diagnostic tests only available in selected centers^93^,^94^,^95^ and it is associated with a large amount of diagnostic and financial overhead. We have shown that additional measures related higher-order, complexity statistics of heartbeat dynamics as well as from their variability over time are the most useful in discriminating PD patients from controls, possibly pointing towards their employment in a lightweight (i.e., ECG-based only) and therefore more widespread diagnostic environment.

Therefore, overall, opposite to the common concept that cardiovascular variability decreases with disease, we have found a measure of variability that increases in the presence of some pathological states, and decreases with states of mental well-being.

Taken together, these evidences suggest that our Lyapunov-based, heartbeat complexity variability measures could be employed as a putative biomarker of psychiatric and/or neurological well-being, where higher complexity variability is associated with a more severe pathological state. In addition, complexity variability might allow for better stratification of pathological subtypes, providing an additional discriminant dimension in building a feature space. These findings are in agreement with the current literature which posits that cardiovascular disorders affect complexity and variability^1^,^9^,^10^,^11^,^12^,^13^,^14^,^15^,^16^. On the methodological side, it is important to mention that our IDLE estimates are not affected by the intrinsically discrete nature of heartbeat dynamics, which are unevenly spaced in time. Additionally, our IDLE estimates are independent of signal background noise statistics^7^,^8^, which have been seen to heavily confound the detection of disease-related alterations in complexity^24^. Of note, we used discrete Laguerre expansions on cubic autoregressive Wiener-Volterra models to achieve long-term memory and improved performances in parameter estimation, as confirmed by goodness of fit measures^7^.

Also, unlike other methods that might require relatively long-term recordings, our method is potentially useful to obtain complexity measures from short recordings. Future work will investigate the potential of these time-varying complexity estimates in producing new real-time measures for the underlying complexity of physiological systems.

## Methods for Cardiovascular Complexity Variability Estimation

In this section, details on the signal processing methodology for the cardiovascular complexity variability estimation are reported. A summary of all indices used in this study is reported in Table 5.

### Lyapunov Exponents Estimation

The Lyapunov Exponent (LE) of a real valued function *f(t*) defined for *t* > 0 is ref. 96:


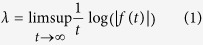


More generally, let us consider *n*-dimensional linear system in the form *y*_*i*_ = *Y(t)p*_*i*_, where *Y(t*) is a fundamental solution matrix with *Y*(0) orthogonal, and {*p*_*i*_} is an orthonormal basis of 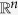. Then, the sum of the corresponding *n* Lyapunov Exponents (*λ*_*i*_) is minimized, and the orthonormal basis {*p*_*i*_} is called “normal”^96^. One of the key theoretical tools for determining LEs is the continuous QR factorization: *Y(t*) = *Q(t)R(t*)^97^,^98^ where *Q(t*) is orthogonal and *R(t*) is upper triangular with positive diagonal elements *R*_*ii*_, *i* = 1:*n*. Therefore we obtain^96^,^97^,^98^:


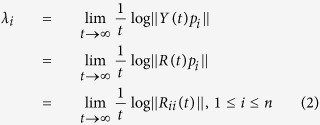


Considering *N* data samples, we evaluate the Jacobian over the time series, and determine the LE by means of the QR decomposition:


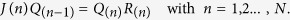


This decomposition is unique except in the case of zero diagonal elements. Then, leveraging on the estimation of the matrices *R*_(*n*)_, the LEs *λ*_*i*_ are given by


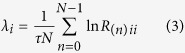


where *τ* is the sampling time step, and *R*_(*n)ii*_ is the value in the diagonal taken by the *i*^*th*^ row and *i*^*th*^ column.

### Nonlinear Modeling of History Dependence

The expected value of a nonlinear autoregressive model can be written as follows:


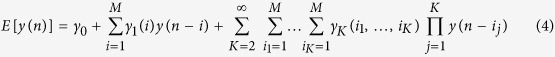


Due to the autoregressive structure of (4), the system can be identified with only exact knowledge of the output data and with only few assumptions on the input data.

An important practical limitation in modeling high-order nonlinearities using the model in (4) is the high number of parameters that need to be estimated from the observed data. An advocated approach to solve such a limitation is the use of Laguerre functions^99^,^100^,^101^,^102^. Let us define the *j*^th^-order discrete time orthonormal Laguerre function:


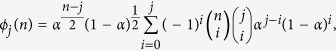


where *α* is the discrete-time Laguerre parameter (0 < *α* < 1) which determines the rate of exponential asymptotic decline of these functions, and *n *≥ 0. Given the Laguerre function, *ϕ*_*j*_(*n*), and the signal, *y(n*), the *j*^th^-order Laguerre filter output is:


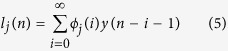


### Time-Varying Modeling of Heartbeat Intervals

The iterative estimation along time of the time-varying complexity and related *complexity variability* index can be performed using several signal processing methods. For example, traditional recursive least-square and window-based methods can be applied. In addition, a simple Kalman filtering can be used to track the complex cardiovascular dynamics at each heartbeat, whereas an instantaneous estimation (i.e., at each moment in time) can be performed using point-process modeling.

A random point process is a stochastic process whose elements are point patterns specified as a locally finite counting measure^103^. Considering the R-waves detected from the Electrocardiogram (ECG) as such events, point process theory can be used to characterize their probability of occurrence^76^,^104^,^105^,^106^,^107^. Mathematically, in the time domain, a simple 1-D point process consists of series of timestamps marking the occurrence times *t* ∈ [0,∞) of the random events. Given a set of R-wave events 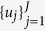, let *RR*_*j*_ = *u*_*j*_*u*_*j*−1_ > 0 denote the *j*^*th*^ R–R interval, or equivalently, the waiting time until the next R-wave event. Assuming history dependence, the probability distribution of the waiting time *tu*_*j*_ until the next R-wave event, where *u*_*j*_ denotes the previous R-wave event occurred before time *t*, follows an inverse Gaussian (IG) model:


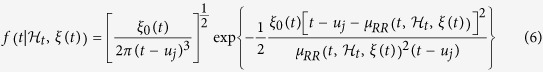


where 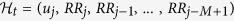 is the history of the point process, *ξ(t*) is the vector of the time-varing parameters, 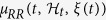 represents the first-moment statistic (mean) of the distribution, and *ξ*_0_(*t*) = *θ* > 0 denotes the shape parameter of the IG distribution (as *θ/μ* → ∞, the IG distribution becomes more like a Gaussian distribution). As 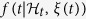 indicates the probability of having a beat at time *t* given that a previous beat has occurred at *u*_*j*_, its first moment 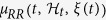 can be interpreted as the average (or expected) waiting time before the next beat. We can also estimate the second-moment statistic (variance) of the IG distribution as 
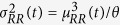
. The use of an IG distribution to characterize the R-R intervals is physiologically motivated: if the rise of the membrane potential to a threshold initiating the cardiac contraction is modeled as a Wiener process with drift, then the probability density of the times between threshold crossings (the RR intervals) is indeed the inverse Gaussian distribution^76^. It is important to note that, when compared with other distributions, the IG model always achieves the best fitting results^105^. The instantaneous RR mean, 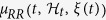, can be modeled as a generic function of the past RR values 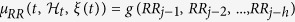, where *RR*_*j*−*k*_ denotes the previous *k*^*th*^ R–R interval occurred prior to the present time *t*.

Here, we represent the nonlinear cardiovascular system by modeling the instantaneous RR mean within a inhomogeneous point-process modeling, taking into account up to the cubic nonlinear terms:


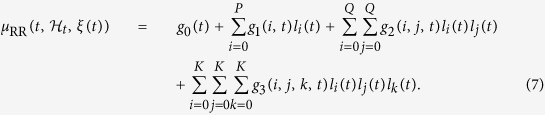


hereinafter called Nonlinear Autoregressive with Laguerre expansion (NARL) model, with *g*_0_,{*g*_1_(*i*)}, {*g*_2_(*i,j*)}, and {*g*_3_(*i,j,k*)} the Laguerre coefficients^7^,^15^.

When *α* = 0 the filter output becomes *l*_*j*_(*n*) = (−1)^*j*^*y(n* − *j* − 1) and the NARL model corresponds, apart for the sign, to the finite nonlinear autoregressive model (NAR) model in (4), whereas for *α* ≠ 0 the instantaneous RR mean is theoretically defined as follows:


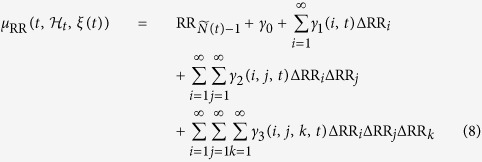


thus having long-term (infinite) memory, and 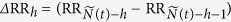. The use of the derivative RR series is aimed at improving model fits in highly non-stationary environments^108^.

Moreover, using the point-process modeling, as 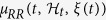 is defined in a continuous-time fashion, we can obtain an instantaneous R–R mean estimate at a very fine timescale (with an arbitrarily small bin size Δ), which requires no interpolation between the arrival times of two beats. Given the proposed parametric model, the nonlinear indices of the HR and HRV will be defined as a time-varying function of the parameters *ξ(t*) = [*θ(t*), *g*_0_(*t*), *g*_1_(0, *t*),..., *g*_1_(*P, t*), *g*_2_(0, 0, *t*),..., *g*_2_(*Q, Q, t*), *g*_3_(0, 0, 0, *t*),..., *g*_3_(*K, K, K, t*)].

A local maximum likelihood method^76^,^109^,^110^ is used to estimate the time-varying parameter set ***x**(t*). We use a Newton-Raphson procedure to maximize the local log-likelihood and compute the local maximum-likelihood estimate of ***x**(t*)^109^. Because there is significant overlap between adjacent local likelihood intervals, we start the Newton-Raphson procedure at *t* with the previous local maximum-likelihood estimate at time *t* − Δ in which Δ define how much the local likelihood time interval is shifted to compute the next parameter update. We determined the optimal orders {*p,q,k*} using the Kolmogorov-Smirnov (KS) statistic in the *post hoc* analysis^76^.

### Calculation of the *Complexity Variability* Index

In our case, the matrix *Y(t*), described in section 5, corresponds to the Jacobian of the *j*-dimensional system of the NARL model parameters, where *j* is the value of the largest lag in the model^6^. Therefore, given the NARL model reported in (7) and using proper transformations^7^, it is possible to obtain an *M*-dimensional state space canonical representation:


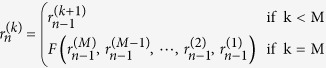


where *F*(⋅) arises directly from (8).

The estimation of the LEs is performed at each time *t* from the corresponding time-varying vector of parameters, *ξ(t*). We define the first LE (*λ*_1_(*t*)) as the instantaneous dominant Lyapunov exponent (IDLE). In this paper, starting from each IDLE series, the time-varying information was condensed by means of the central tendency of the feature distribution, quantified as median (*IDLE*). In addition, we used the Median Absolute Deviation (MAD) with MAD(*X*) = |*X* − Median(*X*)|), of the IDLE to define the *complexity variability* index *CV*_IDLE_.

## Additional Information

**How to cite this article:** Valenza, G. *et al. Complexity Variability* Assessment of Nonlinear Time-Varying Cardiovascular Control. *Sci. Rep.* **7**, 42779; doi: 10.1038/srep42779 (2017).

**Publisher's note:** Springer Nature remains neutral with regard to jurisdictional claims in published maps and institutional affiliations.

## Figures and Tables

**Table 1:** Group Statistics of Features from healthy and CHF subjects.

	CHF	Healthy	p-value
*μ*_*RR*_(ms)	654.77 ± 61.8	863.8 ± 53.7	<4*e*^−4^
*σ*_*RR*_(ms)	8.12 ± 2.0	23.7 ± 7.2	<7*e*^−4^
*LF(ms*^2^)	28.78 ± 19.1	507.3 ± 204.7	<3*e*^−5^
*HF(ms*^2^)	40.29 ± 31.6	627.0 ± 408.2	<1*e*^−3^
*Balance*	0.72 ± 0.4	1.12 ± 0.7	>0.05
*IDLE*	0.0014 ± 0.0649	0.0135 ± 0.0368	>0.05
*CV*_*IDLE*_	0.0595 ± 0.0120	0.0476 ± 0.0066	<0.05

p-values are obtained from the Mann-Whitney test between the CHF and healthy subject groups.

**Table 2:** Group Statistics of Features from the MDD dataset.

	MDD	Healthy	p-value
μRR(ms)	932.96 ± 53.96	921.90 ± 72.57	>0.05
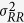 (ms^2^)	1310.70 ± 756.63	958.06 ± 547.60	>0.05
LF(ms^2^)	854.35 ± 672.67	742.52 ± 406.91	>0.05
HF(ms^2^)	1120.38 ± 645.29	906.98 ± 605.64	>0.05
Balance	0.76 ± 0.35	0.78 ± 0.49	>0.05
IDLE	0.035735 ± 0.0405	0.0247 ± 0.0418	>0.05
CVIDLE	0.080021 ± 0.0164	0.0694 ± 0.0149	<0.03

p-value are from the Mann-Whitney non-parametric test with null hypothesis of equal medians. No significant difference was found in any feature except in our novel heartbeat complexity variability index *CV*_*IDLE*_.

**Table 3:** Group Statistics of Features from PTSD before and after Yoga training.

	Before Yoga	After Yoga	p-value
*μ*_*RR*_(ms)	807.27 ± 38.37	789.14 ± 76.85	>0.05
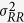 (ms^2^)	207.52 ± 152.96	679.96 ± 601.31	>0.05
*LF(ms*^2^)	573.87 ± 325.02	496.80 ± 274.64	>0.05
*HF(ms*^2^)	213.79 ± 188.52	484.13 ± 459.82	>0.05
*Balance*	2.52 ± 1.82	3.14 ± 2.47	>0.05
*IDLE*	−0.0369 ± 0.0441	0.0036 ± 0.0486	>0.05
*CV*_*IDLE*_	0.0602 ± 0.0139	0.0358 ± 0.0107	<0.02

p-values are from the Wilcoxon non-parametric test for paired data with null hypothesis of equal medians. No significant difference was found in any feature except in our novel heartbeat complexity variability index *CV*_*IDLE*_.

**Table 4:** Statistical analysis between PD and healthy groups.

	PD	Healthy	p-value
*μ*_*RR*_(ms)	915.5 ± 71.9	918.3 ± 101.2	>0.05
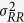 (ms^2^)	203.64 ± 104.84	272.46 ± 117.84	>0.05
*LF(ms*^2^)	184.20 ± 119.13	176.27 ± 108.93	>0.05
*HF(ms*^2^)	121.64 ± 50.50	141.44 ± 78.20	>0.05
*Balance*	1.27 ± 0.80	1.26 ± 0.61	>0.05
*IDLE*	−0.004 ± 0.027	−0.034 ± 0.035	>0.05
*CV*_*IDLE*_	0.0796 ± 0.0140	0.0596 ± 0.0136	<0.05

p-value are from the Mann-Whitney non-parametric test with null hypothesis of equal medians. No significant difference was found in any feature except in our novel heartbeat complexity variability index *CV*_*IDLE*_.

**Table 5:** A summary of all features used in this study.

Feature Symbol	Description	Meaning	References
*μ*_*RR*_	Mean of the Inverse-Gaussian *pdf*	Instantaneous Mean of the RR Interval Series	15,76
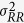	Variance of the Inverse-Gaussian *pdf*	Instantaneous Standard Deviation of the RR Interval Series	15,76
*LF*	Low-Frequency Power of the RR interval series spectrum	Instantaneous Sympathetic and Parasympathetic Activity	15,76
*HF*	High-Frequency Power of the RR interval series spectrum	Instantaneous Parasympathetic Activity	15,76
*Balance*	Ratio between Low- and High-Frequency Power of the RR interval series spectrum	Instantaneous Sympatho-Vagal Balance	15,76
*IDLE*	Dominant (First) Lyapunov Exponent of the RR interval series	Measure of Instantaneous Complexity	7
*CV*_*IDLE*_	Variance of the *IDLE* of the RR interval series	Measure of *Complexity Variability*	7
